# Aggressively progressing primary undifferentiated pleomorphic sarcoma in the eyelid: A case report and review of the literature

**DOI:** 10.1097/MD.0000000000006616

**Published:** 2017-05-05

**Authors:** Won Choi, Jae Ha Hwang, Ga Eon Kim, Kyung Chul Yoon

**Affiliations:** a Department of Ophthalmology and Research Institute of Medical Sciences; b Department of Plastic and Reconstructive Surgery; c Department of Pathology, Chonnam National University Medical School and Hospital, Donggu, Gwangju, South Korea.

**Keywords:** eyelid, rapid progression, undifferentiated pleomorphic sarcoma

## Abstract

**Rationale:**

Undifferentiated pleomorphic sarcoma is extremely rare in the head and neck area, and the aggressive nature of this tumor has not been previously described.

**Patient concerns:**

Therefore, we report a unique case of a very rapidly growing primary undifferentiated pleomorphic sarcoma in the eyelid. A 67-year-old woman presented with a left lower lid mass. She had no previous history of surgery, skin malignancy, or radiation to the eyelid. On initial presentation, the patient showed a 2 × 1 × 1 cm firm, yellow nodule in the left lower eyelid.

**Diagnoses:**

We planned complete surgical removal of the mass; however, the patient was lost to follow-up. One month after the initial visit, the mass had markedly enlarged to 3 × 2.2 × 2.3 cm and a new 2 × 2 × 2 cm mass was observed in the left lower eyelid.

**Interventions:**

Complete surgical resection of the tumor was performed with a myocutaneous free flap. At that time, two masses were emerged, which had grown to 8 × 8 × 5 cm.

**Outcomes:**

The patient died from sepsis caused by pneumonia 2 months after surgery without evidence of local recurrence.

**Lessons:**

Primary undifferentiated pleomorphic sarcoma in the eyelid is extremely rare. Surgeons should be aware of this abruptly presenting, rapidly growing primary eyelid tumor and it is essential to excise the tumor completely as soon as possible.

## 1 Introduction

Sarcomas are malignancies that arise from transformed cells of mesenchymal origin. High grade pleomorphic malignant tumors of the soft tissue that do not differentiate into specific histologic features are classified as undifferentiated pleomorphic sarcoma, previously known as malignant fibrous histiocytoma (MFH). After the World Health Organization (WHO) classification of soft tissue tumors was published in 2002, an extensive literature review identified only few articles describing cases of primary undifferentiated pleomorphic sarcoma of the eyelid.^[1–3]^ However, the authors did not describe how quickly the mass was growing depending on the certain period of time. Furthermore, after the term MFH was completely replaced by undifferentiated pleomorphic sarcoma in the 2013 WHO soft tissue sarcoma classification,^[4]^ there has been no report about primary eyelid undifferentiated pleomorphic sarcoma. Here, we report a case of extremely rare and very rapidly growing primary undifferentiated pleomorphic sarcoma originating from the lower eyelid over a period of 6 weeks.

## 2 Case report

A 67-year-old woman presented to the out-patient clinic with a 3-month history of a left lower lid mass. The patient had a 20-year history of type 2 diabetes mellitus that required insulin, with micro- and macro-vascular complications such as chronic kidney failure and diabetic retinopathy. She was on hemodialysis and also suffered from arterial hypertension and carotid artery disease. She was being treated with amlodipine, valsartan, ferrous sulfate, bisoprolol, clopidogrel, and acetylsalicylic acid. She reported no previous history of trauma, surgery, skin malignancy, or radiation to the eyelid.

**Figure 1:**
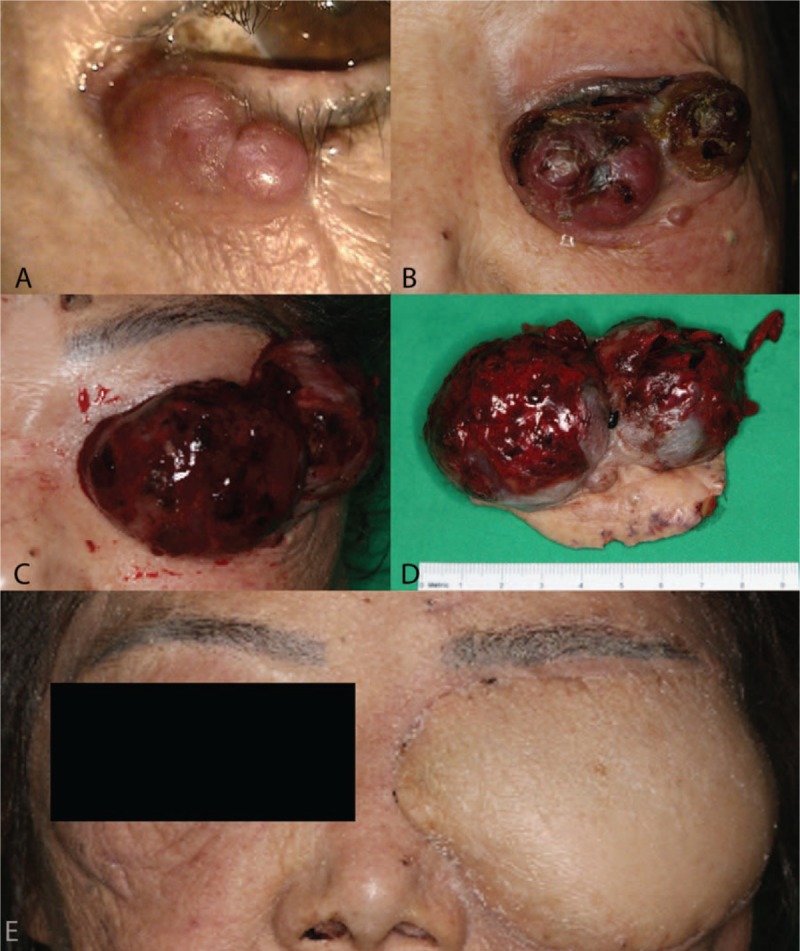
Photographs of a patient with undifferentiated pleomorphic sarcoma of the left lower eyelid at the initial visit (A), 4 weeks after the initial visit (B), 6 weeks after the initial visit (C), and 6 weeks after the operation (D).

**Figure 2:**
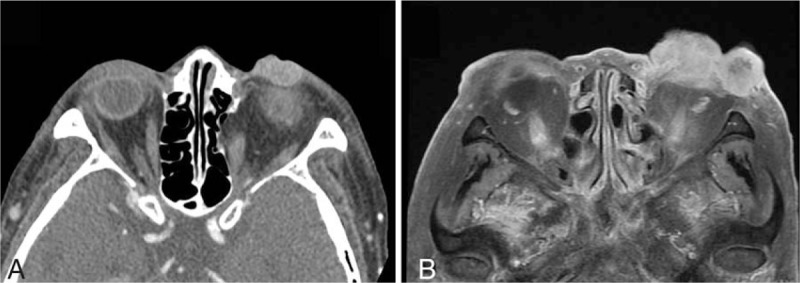
Computed tomography image of the left lower eyelid lesion at the initial visit (A), and a magnetic resonance image 4 weeks after the initial visit (B).

The clinical examination showed a firm and yellow nodule adherent to the adjacent tissue, resembling a sebaceous cell carcinoma in the medial part of the left lower lid (Fig. 1A). We planned surgery for complete excision of the lower eyelid mass and performed computed tomography (CT) before the operation. The CT image showed a frim, yellow 2 × 1.3 × 1.3 cm well-defined, lobulated enhancing mass in the left lower eyelid (Fig. 2A). However, the patient was lost to follow-up after CT scan due to poor compliance.

Four weeks after the initial visit, she visited our hospital again complaining of markedly enlarged lower eyelid mass (Fig. 1B). A magnetic resonance imaging (MRI) scan revealed the lobulated medial lesion had increased to 3 × 2.2 × 2.3 cm and a new 2 × 2 × 2 cm round enhancing mass was observed in the lateral area of the left lower eyelid (Fig. 2B). After assessing the cardiac risk preoperatively and discontinuing antiplatelet therapy, the patient underwent complete surgical excision followed by reconstruction with a free flap of latissimus dorsi myocutaneous graft under general anesthesia. This was 6 weeks after the initial presentation and at that time the 2 masses had merged and grown up to 8 × 5 × 5 cm (Fig. 1C). Macroscopically, the tumor was originated from lower eyelid and did not invade adjacent periosteum or bony structure. The natures of the excised tumor were reddish, hard, dense, and hemorrhagic without evidence of external necrosis (Fig. 1D). Six frozen sections were examined to achieve complete surgical resection of the tumor and were found to have negative intraoperative margins. The patient and her son refused adjuvant radiation therapy. The survival of the graft was excellent at 6 weeks of follow-up (Fig. 1E).

**Figure 3:**
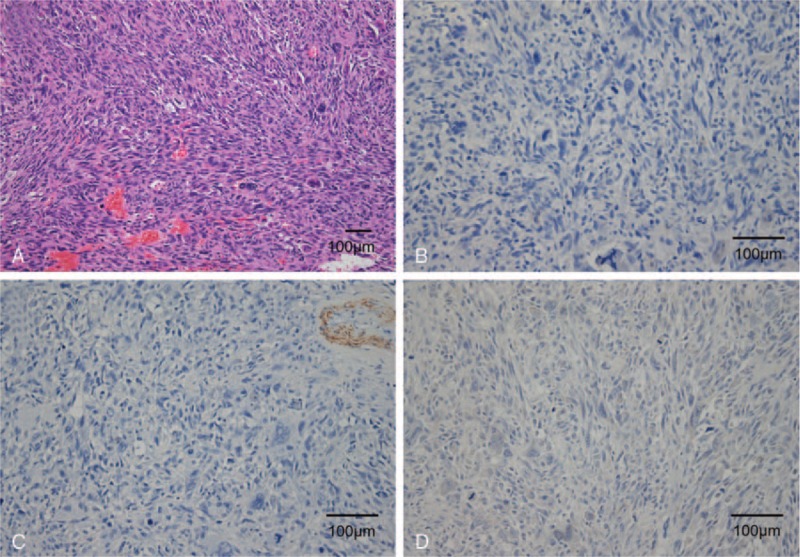
Histopathologic findings. (A) The tumor composed of spindle and pleomorphic cells arranged with fascicular growth pattern. Multinucleated giant cells were observed (Hematoxylin and Eosin). (B) Cytokeratin was negative in the tumor. (C) Smooth muscle actin (SMA) was positive in entrapped normal vessel wall, however, tumor cells were negative for SMA. (D) Immunoreactivity for desmin was not observed.

Histological findings with immunomarkers led to a definitive diagnosis of undifferentiated pleomorphic sarcoma of the lower eyelid. The tumor was composed of fascicular and haphazard arrangement of spindle and pleomorphic cells with eosinophilic cytoplasm and many typical and atypical mitoses. Frequent multinucleated giant cells were present (Fig. 3A). The tumor also showed hemorrhage and focal necrosis. A prominent inflammatory infiltrate, fibrosis, or myxoid change were not found. By immunohistochemistry, some tumor cells were weakly positive for epithelial membrane antigen (EMA), but they were negative for cytokeratin (Fig. 3B), smooth muscle actin (SMA) (Fig. 3C), desmin (Fig. 3D), S-100, Melan-A, and CD34. The tumor did not show a reproducible immunohistochemical profile. The tumor was graded by the French Federation of Cancer Centers Sarcoma Group method and classified as high grade.^[5]^

No metastatic lesions were detected on a positron emission tomography/CT scan. The patient and her son refused further radiation therapy. Two months after the operation, the lesion was stable without evidence of a local recurrence; however, she died from sepsis caused by pneumonia.

This study was approved by the Institutional Review Board of Chonnam National University Hospital. It was in accordance with the principle of the Helsinki Declaration II. Because she died, the written informed consent was obtained from legal guardian.

## 3 Discussion

The 2002 WHO soft tissue sarcoma classification regarded the alternative name of old nomenclature MFH to undifferentiated pleomorphic sarcoma because it provided a more accurate description of the origin of the tumor cells. According to up to date available immunohistochemical techniques, many previously diagnosed MFH tumors have been reanalyzed and only one-tenth of the cases have been rediagnosed as MFH.^[6]^ Furthermore, the category of MFH was completely deleted from the 2013 WHO classification of soft tissue sarcoma. In the head and neck, undifferentiated pleomorphic sarcoma is extremely rare and accounts for only about 3% of all undifferentiated pleomorphic sarcomas.^[7]^ After the 2002 WHO classification, there has been few articles published about a primary undifferentiated pleomorphic sarcoma of the eyelid.^[1–3]^ Hence, our report represents an extremely rare case of a rapidly progressing primary undifferentiated pleomorphic sarcoma of the eyelid after 2002 and 2013 WHO classification.

Our case involved a 67-year-old woman who initially presented to our hospital with a 2 cm^3^ volumed yellow nodule on the lower eyelid. Four weeks after initial visit, she visited again complaining of a marked increase in the size of the previous mass and the appearance of a new round lower eyelid mass. The tumor's volume increased approximately 12 times over just 4 weeks. Furthermore, 6 weeks after the initial visit, 2 masses had emerged and grown to 100 times larger than at the initial visit. Therefore, this is the first report specifically describing the very aggressive nature of undifferentiated pleomorphic sarcoma of the eyelid according to the time passes. The patient underwent complete surgical excision and reconstruction with a free flap of latissimus dorsi myocutaneous graft; however, she died from sepsis caused by pneumonia even though the lesion was stable without evidence of local recurrence.

The primary management of undifferentiated pleomorphic sarcomas in the head and neck is complete surgical resection. The type of surgical resection can be decided by various aspects, which consist of tumor location, size, depth, invasion of adjacent structures, requirement for reconstruction, and the patient's general condition. The excision contains the skin, subcutaneous tissue, and soft tissue or bone adhere to the mass. Positive resection margin is an important predictor of local recurrence and disease-related mortality.^[8,9]^ Neoadjuvant chemoradiation treatment have been investigated at the extremity and retroperitoneal tissue sarcomas during the past 2 decades, however, similar approaches for head and neck sarcomas have been investigated less. Adjuvant radiotherapy is advised for undifferentiated pleomorphic sarcoma. This is based on the findings of previous randomized trials that surgery plus adjuvant radiotherapy reduces the local recurrence rate compared with surgery-only group.^[10,11]^ However, previous studies have shown that adjuvant radiation therapy does not increase overall survival. Chemotherapy is usually reserved for metastatic cases.^[12]^ The most commonly used anticancer drugs are cyclophosphamide, vincristine, and adriamycin. Trials evaluating the advantages of chemotherapy did not help to detect differences in overall survival.^[13]^ In addition, trials often enrolled patients with tumors of various sizes, grades, histologic subtypes, and location, and used different chemotherapy regimens. Therefore, the role of chemotherapy in undifferentiated pleomorphic sarcoma is very limited. In our case, negative intraoperative margins were obtained; therefore, the lesion was stable until she died 2 months after the operation.

The differential diagnosis of undifferentiated pleomorphic sarcoma includes sarcomatoid carcinoma, melanoma, anaplastic large cell lymphoma, and other high grade pleomorphic sarcomas such as leiomyosarcoma, liposarcoma, and rhabdomyosarcoma. The appropriate immunohistochemical cocktail usually resolves any diagnostic dilemmas in the distinction from carcinoma, melanoma, and lymphoma. Sarcomatoid carcinoma can be distinguished from undifferentiated pleomorphic sarcoma by the positive staining result for cytokeratin.^[14]^ Positive results for S-100 and Melan-A stainings can be valuable clues with regard to the differential diagnosis of this tumor from melanoma. In addition, CD30 or CD45 staining by immunohistochemistry provides helpful clues to differentiate undifferentiated pleomorphic sarcoma from lymphoma. Judicious use of additional immunohistochemistry can facilitate the identification of the origin of tumors to rule out pleomorphic leiomyosarcoma (SMA, desmin, and h-caldesmon), pleomorphic rhabdomyosarcoma (desmin), pleomorphic liposarcoma (S-100, SMA).^[9,12,15]^ In our case, some tumor cells were weakly positive for EMA, however, they were negative for cytokeratin, smooth muscle actin, desmin, S-100, melan-A, CD34, etc. Therefore, we could exclude such entities and finally diagnosed as undifferentiated pleomorphic sarcoma. These are summarized in Table 1, including our case.

**Table 1:**
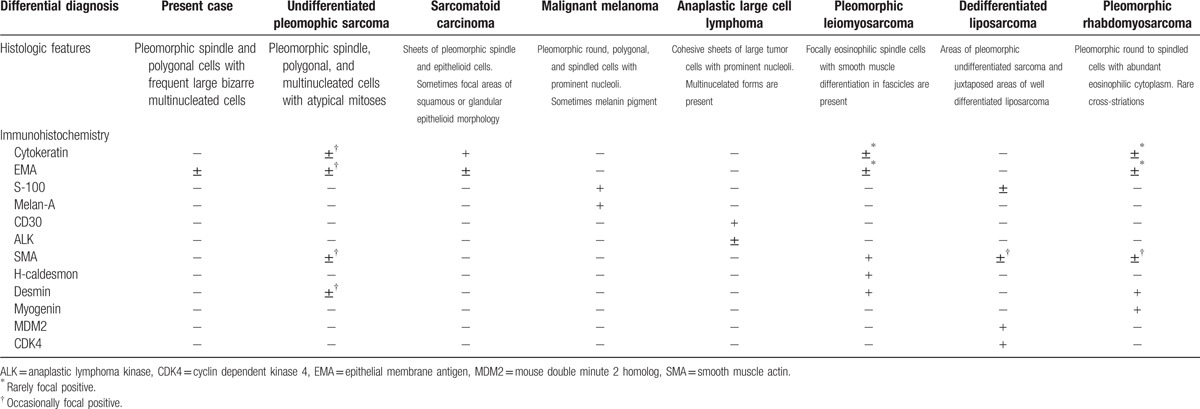
Histological features and immunohistochemical panel to differential diagnosis of undifferentiated pleomorphic sarcoma.

According to the literature, the 5-year overall survival rate for head and neck undifferentiated pleomorphic sarcoma was known as 50% to 60%.^[16]^ On the other hand, trunk and extremity tumors were 73%, which indicated better prognosis than head and neck undifferentiated pleomorphic sarcoma.^[17]^ This result could be explained by higher grade tumors and difficulties to obtain negative tumor margins due to adjacent important structures in the cases of head and neck cancer. At initial presentation, size larger than 5 cm, high histologic grade, presence of metastases were related to a poor prognosis.^[18–21]^ In our patient, the tumor was greater than 5 cm and high histologic grade, however, since she died at 2 months after operation due to sepsis originated from pneumonia, there are limitations for analyzing prognostic factors.

In our case, periocular undifferentiated pleomorphic sarcoma was small, nodular, and located superficially at initial presentation similar to the previous reports. The tumor was located at the medial aspect of lower eyelid. Previously reported eyelid MFH did not show a tendency of location at the eyelid (upper eyelid, medial, and lateral canthus).^[2,3]^ In the previous case report series, all cases underwent local excision as initial treatment and additional wide resection with adjuvant radiotherapy were used for subsequent local recurrence.^[3]^ While all of these cases were high grade and had local recurrence, it is notable that no patient has died as a result of the tumor until several years after operation. This could also be a result of the anatomic location of periocular undifferentiated pleomorphic sarcoma, which leads to earlier presentation, as compared with undifferentiated pleomorphic sarcoma arising in deeper soft tissue.

Our case is important for several reasons. First, this is an extremely rare case of primary undifferentiated pleomorphic sarcoma of the eyelid that was progressing very rapidly as we presented with time passes. Second, this case highlights the necessity of a prompt diagnosis and immediate surgical intervention if clinically suspected. Therefore, it is necessary to keep the possibility of rapid progression in mind for plastic surgeon and ophthalmologists in the cases of clinically suspected as undifferentiated pleomorphic sarcoma.
